# Pathways to the persistence of drug use despite its adverse consequences

**DOI:** 10.1038/s41380-023-02040-z

**Published:** 2023-03-30

**Authors:** Gavan P. McNally, Philip Jean-Richard-dit-Bressel, E. Zayra Millan, Andrew J. Lawrence

**Affiliations:** 1 School of Psychology, UNSW Sydney, Sydney, NSW 2052 Australia; 2 Florey Institute of Neuroscience and Mental Health, Parkville, VIC 3010 Australia; 3 Florey Department of Neuroscience and Mental Health, University of Melbourne, Melbourne, VIC 3010 Australia

**Keywords:** Neuroscience, Addiction

## Abstract

The persistence of drug taking despite its adverse consequences plays a central role in the presentation, diagnosis, and impacts of addiction. Eventual recognition and appraisal of these adverse consequences is central to decisions to reduce or cease use. However, the most appropriate ways of conceptualizing persistence in the face of adverse consequences remain unclear. Here we review evidence that there are at least three pathways to persistent use despite the negative consequences of that use. A cognitive pathway for recognition of adverse consequences, a motivational pathway for valuation of these consequences, and a behavioral pathway for responding to these adverse consequences. These pathways are dynamic, not linear, with multiple possible trajectories between them, and each is sufficient to produce persistence. We describe these pathways, their characteristics, brain cellular and circuit substrates, and we highlight their relevance to different pathways to self- and treatment-guided behavior change.

## Introduction

In 2016, Pickard and Ahmed posed the ‘puzzle of choice’ [1]. They began by considering evidence that people with problematic drug use can frequently control their behavior and can choose to abstain under many circumstances. Given this evidence for controlled drug use, Pickard and Ahmed asked why do these individuals choose to use despite the portent of negative consequences from that use?

The apparent insensitivity of drug taking to its adverse consequences is among the most pernicious features of addiction. It is responsible for a large part of the human toll of addiction, contributing directly to the detriments in health and well-being suffered by individuals, and imposing substantial burdens upon families and communities. Insensitivity to adverse consequences is not limited to drug use. It is shared with other problematic behaviors such as problem gambling that impose real and significant costs on individuals and the community.

Given the key role that insensitivity to adverse consequences plays in the presentation, diagnosis, and impacts of persistent drug use, as well as evidence that the eventual recognition and appraisal of these adverse consequences contributes to decisions to reduce or cease use [2–4], it is not surprising that it has been of intense interest. However, the most appropriate ways of conceptualizing this insensitivity remains unclear [5] and sometimes controversial [6, 7]. The diverse conditions under which insensitivity is observed and heterogeneity in its presentation imply that it is multifactorial. Yet, often only single mechanism solutions are presented for this problem [7], and these are typically presented as linear transitions or trajectories.

**Fig. 1:**
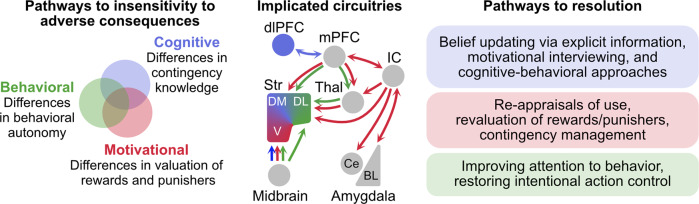
Pathways to insensitivity and their potential resolution. At least three pathways, representing distinct psychological factors, may each be sufficient to produce insensitivity to punishment and cause persistent, detrimental behavior. Insensitivity can arise from poor instrumental contingency knowledge (cognitive pathway), distortions in positive and/or negative valuation of consequences (motivational pathway), and/or alterations in behavioral autonomy (behavioral pathway). These pathways occur against complex backgrounds of intoxication, history of dependence, acute or protracted withdrawal, and acute as well as chronic stressors that can influence capacity to detect, appropriately learn about, weight, and respond to negative consequences. These pathways can operate independently but may also interact. They are underpinned by still poorly understood distinct (as shown in colored) but partially overlapping (as shown in gray) neural circuitries. These pathways are likely dynamic and non-linear. Each may operate within the same individual at different times. Resolution of problematic behaviors will depend on which of these factors is contributing at specific times to cause persistence of behavior. dlPFC—dorsolateral prefrontal cortex, mPFC—medial prefrontal cortex, IC—insular cortex, Thal—thalamus, Str—striatum, DM—dorsomedial striatum, DL—dorsolateral striatum, V—ventral striatum, Ce—central amygdala, BL—basolateral amygdala complex.

Here we argue for three pathways to insensitivity that may contribute to persistent drug use despite its negative consequences (Fig. 1)^1^. A cognitive pathway for recognition of adverse consequences, a motivational pathway for valuation of these consequences, and a behavioral pathway for responding to these consequences. These pathways can operate in the same individual at different times, they can be independent with multiple possible trajectories between them, and each is sufficient to produce insensitivity. These pathways are embedded in complex backgrounds of intoxication, history of dependence, acute or protracted withdrawal, and stress. We describe how these pathways differ from each other and we consider their relevance for self- and treatment-guided behavior change.

## The adverse consequences of drug use

Although not all drug use, even when prolonged, has negative consequences for an individual, prolonged drug use can have profound deleterious impacts on the health and well-being of individual users, ranging from acute toxicity to chronic health conditions [8–10]. Some adverse consequences may be directly, causally linked to drug use (e.g., overdose, hepatitis, HIV), whereas others are multifactorial and drug use adds to their risk (e.g., psychiatric disorders, cancers). Regardless, the burden of illicit drug use is estimated at 0.8% of global all-cause disability adjusted life years [9, 11]. The impact of licit substance use is greater still with alcohol use alone accounting for 5.1% of the global burden of disease and injury [12]. Adverse effects are not restricted to impacts on health. Persistent substance use creates and exacerbates relationship and interpersonal problems and can lead to detrimental neglect of familial and occupational responsibilities as well as direct harm to others. Persistence of use in the face of adverse impacts is held to distinguish problematic from recreational drug use. It also forms a key part of formal diagnosis [13, 14].

Non-human animal models of drug use serve an important role in studying adverse consequences because they allow precise control over drug history, assessment of individual differences, and titration of adverse consequences. Indeed, there has been progress in understanding whether, when, and why laboratory animals persist in drug self-administration despite adverse consequences [15]. In a typical experiment, rats or mice are first trained to self-administer a substance, under simple (e.g., fixed ratio) or more sophisticated (e.g., ‘seeking-taking’) schedules. Then, adverse consequences are introduced to punish this behavior, such as arranging that responding for the substance also leads to footshock [16–20], conditioned fear [21, 22], air puffs [23], or adulteration of the substance [24, 25]. The precise parametric relationship between drug seeking or taking and punishment varies across studies and these parameters are important to consider because they constrain and confound interpretation [19, 26]. In general, though, under these conditions, some laboratory animals reduce or cease drug self-administration, showing that drug seeking and taking are sensitive to adverse consequences. However, despite being subjected to negative consequences, some laboratory animals persist in seeking and taking. Such persistence has been observed in laboratory animals self-administering cocaine [19, 27–31], heroin [32], methamphetamine, and alcohol [24, 33].

This work has identified at least five features of insensitivity to adverse consequences. First, when low to moderate intensity punishers are used, only some mice [24] and rats [20, 33] are insensitive, speaking to individual variability in humans. Second, factors relating to initial drug use, including preference, intake, and rate of self-administration, are unrelated to insensitivity [18, 19, 21, 29, 34, 35]. This shows that insensitivity is separate to mechanisms governing initial use [36–38]. Third, extended self-administration and longer access per self-administration session favor insensitivity [19, 27, 38]. Fourth, propensity to drug seek under progressive ratio schedules [16, 18–20, 28, 30, 33, 39, 40], increased responding during extinction [18, 21, 28, 39, 40] or increased economic demand [41, 42] can predict insensitivity (but see [29, 35, 43–45]). Fifth, response impulsivity, such as reduced ability to wait before performing an appropriate response [29, 35] can predict insensitivity. These features are robust and replicable. They are important because they speak to, and constrain, mechanisms for insensitivity. We draw on these features when considering properties of the three putative pathways to insensitity to adverse consequences.

## Cognitive pathways: recognizing adverse consequences

The cognitive pathway to persisting in drug taking despite negative consequences refers to the knowledge that an individual has about the consequences of their drug use (Fig. 1). Recognizing and identifying the negative consequences of drug use are necessary conditions for those consequences to change behavior. Yet for many individuals, choices to use a drug often do not incorporate possible adverse consequences from that use [3, 4, 46]. To be sure, severe adverse consequences such as financial, familial, medical, employment, or legal problems can predict treatment seeking in some people [47, 48]. However, recognition of the severity of these consequences and their relationship to drug use is rarely immediate [1]. Recognition that one’s drug use is responsible for negative consequences often occurs gradually, with individuals engaging in cognitive appraisals that can help drive self- or treatment-guided behavior change [2–4, 49, 50].

Engaging in these reappraisals may be difficult. Counselling approaches, such as motivational interviewing, capitalize on and enhance motivation to change [51]. These approaches can be highly effective for some people [52] but they can be less effective at creating the initial motivation to change [51, 53]. Reappraisals start gradually and behavior changes irregularly, often in highly personalized ways [3, 49]. This individuality has been argued to be beyond the reach of neuroscience [3], but recent work is beginning to show why correctly recognizing the negative consequences of our actions can be difficult.

Experience does not always deliver veridical causal knowledge about our actions. Humans [54–56] and other animals [57, 58] differ profoundly in what they learn about the consequences of their actions. These differences in learning can drive pronounced differences in choice. Some people form correct causal beliefs about how their actions cause adverse consequences. They can use this knowledge to choose other behaviors. In contrast, other people form incorrect beliefs about the causes of adverse consequences. Their lack of correct awareness leads them to choose actions with adverse consequences.

For example, Jean-Richard-dit-Bressel et al. [54] used a conditioned punishment task in young adults that allowed them to choose between two responses, one earning probabilistic rewards and one earning probabilistic reward and punishment. Participants were provided with no information about the specific punishment contingencies. They had to learn these from experience. Some readily learned to reallocate behavior away from the punished behavior, others did not. These differences were not due to differences in engagement with the task or in valuation of reward or punishment. Instead, sensitive and insensitive individuals differed profoundly in what they learned. Punishment sensitive individuals acquired correct causal beliefs about their behavior. They learned the correct Action–Punisher contingencies that they used to avoid further punishment. Punishment insensitive individuals also learned Action–Punisher contingencies. However, what they had learned was incorrect. Insensitive individuals formed incorrect beliefs about the causes of negative consequences so they could not withhold the specific action that caused punishment. This same bimodal punishment sensitivity driven by deficits in accurate instrumental contingency knowledge is observed in non-human animals [57], suggesting that it is a core property of learning and amenable to mechanistic deconstruction.

Lacking awareness or possessing erroneous causal beliefs about the adverse consequence of a behavior is not always problematic. In many individuals, lack of awareness or incorrect causal beliefs can be corrected to change behavior [59]. For example, explicit information about Action–Punisher contingencies changes the behavior and beliefs of some insensitive people, causing them to cease that behavior and avoid further punishment. However, lack of awareness or incorrect beliefs can sometimes be problematic. In some insensitive people, behavior resists counterevidence about why they are being punished, trapping them in a cycle of repeating negative consequences [59]. This is likely under at least one condition: when adverse consequences are infrequent [59]. Under this condition, some insensitive individuals discount veridical counterevidence about the causes of punishment and detrimental behavior persists [59].

So, insensitivity to adverse consequences can emerge from the different things that we learn about the negative consequences of our actions. Three features make the negative consequences of drug use especially prone to this insensitivity. First, the negative consequences of drug use are probabilistic, and any experienced contingencies are typically weak. The probability that any individual act of drug use will have detectable negative consequences for the individual is low. This makes learning about adverse consequences from experience difficult. We underweight rare, adverse events when making experience-based choices [55, 60]. Indeed, when individuals do experience adverse consequences from initial drug use (e.g., nausea in response to nicotine; flushing in response to ethanol), further use can be slowed [61–64]. Second, the trajectory of drug-related harms often involves escalation from minor to more severe, typically over many years. This trajectory undermines the ability of those consequences to change behavior. Severe negative consequences are less effective at changing behavior if they have been preceded by less severe negative consequences than if they had been experienced from the outset [65–67]. Third, the negative consequences of drug use are often temporally removed from the act of use. This delay between cause and effect further undermines learning [68, 69] and the capacity of negative outcomes to shape choices and behavior.

The brain mechanisms underlying this cognitive pathway are poorly understood. Core features are that valuation and action control can be intact but individuals differ in correct awareness of the consequences of their behavior as well as in their willingness to update beliefs in response to counterevidence. Progress in understanding may benefit from considering theoretical and computational advances in the mechanisms of belief updating [70–73] and their application to neuropsychiatric conditions [74–76]. This has identified key roles for dopamine [77] and a network of cortical regions involving dorsolateral prefrontal cortex and their interactions with medial prefrontal cortex [78, 79]. Interestingly, these mechanisms for belief updating depend on more than just the prediction error often studied in addiction neuroscience. For example, they depend on the meaningfulness of the new information being considered to the belief rather than on just how surprising or different from expectation that information is [78]. There is some evidence from laboratory animal studies that exposure to addictive drugs may alter these updating processes [76].

Given these pronounced individual differences in what we learn about the adverse consequences of behavior and in our willingness to change behavior in response to counterevidence, we argue that a cognitive pathway to persistent drug use despite potential negative consequences is more common than appreciated in the addiction neuroscience literature. Correctly recognizing the adverse consequences of one’s actions is more complex than simply experiencing or being educated about those consequences. Sustaining correct recognition may be equally difficult [75, 76]. These cognitive barriers must be overcome if negative consequences are to shape future choice and action.

## Motivational pathways: valuing adverse consequences

If the negative consequences of drug use have been recognized and attributed, then a second pathway to persisting in drug taking despite adverse consequences can be linked to distortions in value-based choice [6, 80, 81] (Fig. 1). This has emerged from literature studying drug choice and economic demand [6, 41, 80, 82, 83] (see [6, 84, 85] for review). It shows that drug seeking and taking can be strongly linked to the relative value of the drug which, in turn, is determined by its relevance to the users’ current desires and needs. Drugs can be chosen when their expected benefits exceed those of other behaviors, and they exceed any expected costs. When controlled by expected value, drug use can be highly flexible, with individuals choosing to use despite negative consequences and choosing to abstain should there be sufficient incentive [1, 86, 87]. The evidence for this is compelling [6, 82, 88, 89]. It aligns with laboratory findings from smokers [90], opioid users [85, 91] and polydrug users [92] as well as with self-reports that cognitive appraisals and cost-benefit evaluations about use precede self- and treatment-guided behavior change [2–4, 49, 50]. It also aligns well with the evidence that cognitive re-evaluations of the ‘pros and cons’ of drug use are central to behavior change [93].

Pharmacotherapies designed to manage craving and withdrawal as well as treatments such as contingency management capitalize directly on this role of value to provide approaches effective for some people [94]. For example, in voucher-based reinforcement therapy, individuals earn vouchers and other incentives if they reach an agreed therapeutic goal. Success of these treatments is influenced by variables known to influence value computations, including voucher value and immediacy of voucher receipt [94].

Value is pleiotropic. So, distortions of value not only explain why an individual persists in drug-seeking despite adverse consequences but also why this persistence is expressed in other behaviors such as increased break points [16, 18–20, 28, 30, 33, 39, 40], increased responding during extinction [18, 21, 28, 39, 40] and increased economic demand [41, 42] in animal models, highlighting the need for such assessments when attempting to isolate causes of punishment insensitivity. Moreover, this pathway predicts that sensitivity or insensitivity to punishment depends on the experiences of the individual [66, 67, 95].

The importance of value in dictating choices to abstain or use underscores the need for deeper understanding of how negative and positive value are computed and used. One possibility is that excessive valuation is due to increased dopaminergic neurotransmission [96, 97]. To study this, Lüscher and colleagues developed an optical model of midbrain dopamine neuron self-stimulation. Here mice respond under simple [35] or seeking-taking [23] schedules for dopamine neuron excitation that also yield footshock punishment. Under these conditions some mice are insensitive to punishment. This insensitivity is due to increased excitability of orbitofrontal cortex neurons [35] and potentiation of orbitofrontal synapses in dorsal striatum [23, 98]. Remarkably, artificially sculpting plasticity in this orbitofrontal projection induced persistent seeking in punishment sensitive mice and reversed persistent seeking in punishment resistant mice [98]. Whether plasticity in this circuit adjusts response and/or outcome values remains unclear. Furthermore, the mechanisms for valuation are likely to be more complex still. Dopamine serves different roles in learning depending on its specific local and long-range circuit features [99–101]. This role is often value free [102, 103] and increasing dopamine neurotransmission has also been shown to increase, not decrease, punishment learning [104].

A second possibility is linked to the repeated cycles of instrumental incentive learning [105, 106] embedded in drug use. Here, individuals learn from experience that drug taking augments positive emotional states and/or ameliorates negative ones (boredom, social exclusion, depressed mood, drug withdrawal) [107–110]. This learning causes a revaluation of the drug, inflating its value and transforming it into a goal to be sought in future such states. For example, experiencing the alleviation of drug withdrawal by drug intake increases the incentive value of the drug in, and propensity to seek and take the drug during, future withdrawal states. This learning is outcome (i.e., drug) and state specific, so it can account for context-specific drug preferences and their reversal in different settings [111–114]. Incentive learning is well established for non-drug reinforcers [105, 115, 116]. However, evidence from drug reinforcers is sparse [117]. Understanding whether this incentive learning guides drug choices in the face of adverse consequences remains important, as does understanding how pharmacotherapies for withdrawal management affect this transformation and use of drug values.

The neurobiological mechanisms for these choices to seek and consume drugs despite negative consequences have attracted considerable attention. In addition to the mechanisms described above, insula cortex, basolateral amygdala and their interactions are essential to encoding internal states [118] and dynamic changes in reward and punishment value in humans, non-human primates, and rodents [106, 119–125]. Meta [126] and mega [127] analyses show alterations in human insula volume and/or gray matter thickness across dependence to several drugs [128], that could suggest potential alterations in punishment or reward encoding. This is supported by alterations in resting state functional connectivity of frontal and insular cortical regions in cocaine users [129].

Laboratory animal studies extend these findings. Propensity to seek alcohol [130, 131] or nicotine [132] after punishment or seek methamphetamine after voluntary (i.e., choice-induced) abstinence [133] is associated with increased activity in the anterior insula whereas choice-induced prevention of incubated craving for methamphetamine is linked to reduced anterior insula cortex activity [134]. Studies with non-drug rewards implicate insula projections to ventral striatum, particularly accumbens core, in retrieving and using outcome values in action selection [135, 136]. This pathway also mediates punishment resistant alcohol drinking [25]. However, other insula projections are relevant, including projections to the central amygdala. This projection mediates propensity to choose methamphetamine when alternative non-drug choices are removed [133]. Activity of amygdaloid PKCδ + neurons predicts punishment resistant alcohol drinking [33]. Reducing activity of these neurons reduces punished alcohol choice [33] and mediates the protective effect of non-drug choices on incubation of methamphetamine craving [134]. However, this circuitry is more complex still, with important roles for orbitofrontal cortex [137, 138]. Both lateral [139] and medial [140] orbitofrontal cortex are essential for learning about the adverse consequences of behavior. Studies with non-drug rewards show that distinct orbitofrontal cortical-amygdala projections in encoding (lateral orbitofrontal → basolateral amygdala) and retrieving (medial orbitofrontal → basolateral amygdala) reward value [138]. The role of these orbitofrontal cortical-amygdala interactions in choices to seek and take drugs despite negative consequences are important issues for the field to address.

Medial prefrontal cortex and its projections are also relevant. Medial prefrontal cortex is essential to value-based choice [141, 142] including cost-benefit decisions [143]. Medial prefrontal cortex shows structural alterations across dependence to several drugs [126, 127] and animal studies show that drug self-administration and exposure remodels [144] and reduces excitability [24, 145] as well as plasticity [16, 28] of prefrontal neurons. These circuits, especially those in the rodent prelimbic cortex and its projections to striatum and midbrain, are directly implicated in the choice of reward (sucrose, alcohol, cocaine) under punishment [16, 24, 28, 146–148]. Moreover, punishment resistant seeking has been linked to reductions in excitability of prelimbic neurons, while increasing activity in these prelimbic circuits can reduce choice of punished cocaine [148] and alcohol [24].

Choice is more than behavioral allocation. Process models such as sequential sampling models (e.g., drift-diffusion [149] and linear ballistic accumulator [150]) provide computationally tractable decomposition of choice into its latent cognitive processes [37, 151–153]. These models identify computational similarities [154] between human [151], non-human primate [151], and rodent [155] choice. They have parallels to circuit function [156–160], holding promise for achieving a formal understanding of how value is used when making drug choices [81, 161], complementing reinforcement learning models for learning this value [149, 162]. They may help identify how medications and interventions facilitate cognitive appraisal of the negative consequences of use that drive abstinence. Field et al. [80] have shown that these process models provide coherent explanations of drug choices as well as their remediation across recovery (see also [81]). This aligns with demonstrations that deliberative choice, including when evaluating the risk versus benefits of seeking rewards under punishment, is linked to medial prefrontal cortex [155, 163, 164] and its projections to thalamus [155, 165]. These findings are relevant to evidence that training deliberative choice of non-alcohol rewards over alcohol reduces rates of relapse to alcohol drinking in the high-risk period following inpatient discharge [166]. Process models also hold promise for understanding intra-individual variation in choice. Even with correct understanding of consequences, choices are not always optimal and preference is not always stable. Process models provide one way of understanding stochastic and probabilistic variation in choice including those based on fluctuations in value and cognitive control.

## Behavioral pathways: responding to adverse consequences

If the negative consequences of drug use have been recognized, attributed, and valued, then a third pathway to persisting in drug taking despite adverse consequences is behavioral (Fig. 1) [37, 38, 167, 168]. Proposed and elaborated by Everitt, Robbins et al. (see [167, 169, 170] for review), this pathway is based on dichotomy of control by goal-directed versus habitual instrumental learning [171, 172] and their distinct neural circuit bases [173–180]. Across prolonged drug self-administration, there may be a transition from intentional control of value-based choice to Stimulus–Response control that is separate to any value of the drug to the users’ needs or desires. Such seeking is a relatively automatic response to antecedent environmental and behavioral stimuli [181].

Drug seeking as a habit may be autonomous but not necessarily insensitive to adverse consequences. Stimulus–Response associations are not immutable and they may be especially fragile [182]. For example in the laboratory, detection of behavioral autonomy depends on context [183] and number of behavioral choices [184], among other variables [185]. Thus, it is additionally assumed that a feature of drug seeking Stimulus–Response habits, and a characteristic that makes them resistant to their adverse consequences, is that they are divorced from the reinforcement mechanisms that would otherwise update them [167]. Under these conditions, drug seeking can be insensitive to any consequences, adverse or otherwise [167, 169].

The relevance of this pathway to the experiences and behavior of drug users has been questioned [6, 86, 87, 186]. This pathway may be the most difficult to study because it requires exclusion of the cognitive and motivational pathways [19, 29, 34, 43–45, 187]. Nonetheless, this pathway captures a core feature of human and other animal behavior [188]. Choices, including among cocaine [189] or alcohol [190] dependent individuals, can sometimes be independent of the current value of what is being chosen [171, 191, 192]. The behavioral pathway offers parsimonious explanation of examples of drug seeking in humans (e.g., absentminded relapse) [7, 193] that are reminiscent of the ‘slips of action’ observed in human laboratory choice tasks [190, 194, 195].

The brain mechanisms of this behavioral pathway are based on findings in humans and rodents identifying a change in control from ventral to dorsal and then from dorsomedial to dorsolateral striatum/putamen, as well as a reduction in “deliberative” medial prefrontal control, as behavior becomes more stimulus-bound [36–38, 167, 169, 194, 195]. They include, but are not limited to, demonstrations from laboratory animals that prolonged self-administration promotes dorsolateral striatal control of cocaine [196], alcohol [197–200], and heroin [201] seeking. Importantly, the control of choice by Stimulus–Response mechanisms in alcohol-dependent individuals is associated with increased putamen choice-related activity [190]. The cortical morphological and volumetric changes seen in drug dependence [127, 128] are obvious candidates for alterations in top-down control. Indeed, choice in alcohol-dependent individuals is associated with reductions in ventromedial prefrontal cortex activity [190].

Punishment-resistant drug seeking can depend on these striatal circuits. For example, Jonkman [202] showed that cocaine seeking under punishment, but not in the absence of punishment, was reduced by reversible inactivation of dorsolateral striatum. Giuliano et al. [187] extended this to show that persistent alcohol seeking despite punishment was predicted by the extent to which alcohol seeking depended on dopamine receptors in the dorsolateral striatum. Crucially, after rats had been identified as sensitive or insensitive to punishment, dorsolateral striatal dopamine receptor antagonism only reduced seeking in laboratory animals insensitive to punishment. Giuliano et al. could exclude differences in propensity to drink alcohol, differences in alcohol preference, and differences in alcohol self-administration as causal to this insensitivity. Persistence of alcohol-seeking in the face of punishment may be due to this failure to disengage dopamine-dependent signalling in the dorsolateral striatum. The mechanisms for this are poorly understood but these are important targets for pharmacotherapies due to their potential to facilitate value-based choice essential for cognitive appraisals of drug use. It is worth noting that a common role for dorsolateral striatal circuitry in punishment-resistant drug seeking and a role for this circuitry in autonomous Stimulus–Response associations does not show that such associations drive punishment resistance, but it does add some evidence for this behavioral pathway.

Alterations in endocannabinoid [203] and serotonin signalling (5-HT) may also be relevant. 5-HT has complex roles in behavioral control [204] that may contribute to insensitivity. Pelloux et al. [31] showed that rats insensitive to punishment of cocaine seeking under a seeking-taking schedule had reduced 5-HT turnover in prefrontal cortex and striatum. Insensitivity was alleviated by the selective serotonin-reuptake inhibitor citalopram [31]. This was linked to 5-HT actions at 5-HT_2C_ receptors because 5-HT_2C_ agonist mCPP counteracted insensitivity while 5-HT_2C_ antagonist increased punished cocaine-seeking. Other 5-HT receptors have also been implicated. Lüscher and colleagues showed that 5-HT_1B_ receptors on orbitofrontal cortical projections to dorsal striatum promote presynaptic depression of this projection and maintain sensitivity of cocaine taking to punishment in mice [205]. Projection-specific knock-out of these 5-HT_1B_ receptors reduced the sensitivity of cocaine taking to punishment [205]. These findings suggest that upregulating 5-HT may assist in pharmacotherapy for drug use disorder, but evidence remains unclear [206].

## Conclusions

The persistence of drug use despite negative consequences is complex. We have argued that this is not a unitary construct but rather that there are at least three pathways to this persistence—cognitive, motivational, and behavioral. These pathways are dissociable but they are neither mutually exclusive nor exhaustive. They may operate dynamically within the same individual at different times. They can also interact. For example, valuation (motivational pathway) depends on correctly recognizing actions and their consequences (cognitive pathway) but valuation also governs salience and detection of these consequences [207, 208]. Likewise, transient fluctuations in attention to action can influence relative contributions of the cognitive or behavioral pathways [183]. Crucially, these pathways occur against complex backgrounds of intoxication, individual histories of drug use, acute or protracted withdrawal, and acute as well as chronic stressors that can influence capacity to detect, appropriately learn about, weight, and ultimately respond to negative consequences.

Different pathways for persistent drug use despite adverse consequences align well with and may help reconcile findings that there are only partially overlapping brain circuitries for this persistence. We argue that understanding how insensitivity to adverse consequences arises has important implications not only for understanding the underlying brain mechanisms of this persistence but also for understanding how different pharmacotherapies and treatment strategies may act, possibly in complementary ways, to reduce this persistence and enhance sensitivity to adverse consequences.

Finally, different pathways for persistent drug use despite its adverse consequences align well with the fact that recovery from drug addiction is highly personalized. Choices to use or abstain from use of a drug are not immalleable. Individuals follow different pathways to self- or treatment-guided recovery—where the necessary appraisals of costs versus benefits of use are predicated upon being able to recognize those costs, evaluate their importance, and adjust behavior in response to them. Progress is neither linear nor always predictable. We argue that resolution of problematic behaviors will depend on which of these pathways is contributing at specific times to cause persistence of behavior. A better understanding of why behaviors persist despite adverse consequences, and a more thorough examination of these underlying pathways to insensitivity may help us understand these recoveries, improve understanding of the variation in efficacy of existing treatment strategies, as well as promote development of more effective individualized treatments.

## Footnotes

1 We use the terms ‘punishment insensitivity’ and ‘persistence despite negative consequences’ in preference to the term ‘compulsivity’. The studies we review operationalize insensitivity to adverse consequences via punishment procedures and our focus is on how people and other animals may fail to detect, learn, and respond to these adverse consequences. The reader is referred to George et al. [5] for evaluation of use of the term ‘compulsivity’ in addiction neuroscience.
